# Quality of care for the treatment for uncomplicated malaria in South-East Nigeria: how important is socioeconomic status?

**DOI:** 10.1186/s12939-015-0150-6

**Published:** 2015-02-14

**Authors:** Ogochukwu P Ibe, Lindsay Mangham-Jefferies, Bonnie Cundill, Virginia Wiseman, Benjamin S Uzochukwu, Obinna E Onwujekwe

**Affiliations:** Health Policy Research Group, Department of Pharmacology and Therapeutics, College of Medicine, University of Nigeria, Enugu, Nigeria; Department of Global Health and Development, London School of Hygiene and Tropical Medicine, London, UK; Department of Infectious Disease Epidemiology, London School of Hygiene and Tropical Medicine, London, UK; Department of Community Medicine, College of Medicine, University of Nigeria, Enugu, Nigeria; Department of Health Administration and Management, Faculty of Health Sciences, University of Nigeria, Enugu, Nigeria

**Keywords:** Malaria, Inequity, Socioeconomic status, SES, Artemisinin combination therapy, Nigeria

## Abstract

**Introduction:**

Ensuring equitable coverage of appropriate malaria treatment remains a high priority for the Nigerian government. This study examines the health seeking behaviour, patient-provider interaction and quality of care received by febrile patients of different socio-economic status (SES) groups.

**Methods:**

A total of 1642 febrile patients and caregivers exiting public health centres, pharmacies and patent medicine dealers were surveyed in Enugu state, South-East Nigeria to obtain information on treatment seeking behaviour, patient-provider interactions and treatment received. Socioeconomic status was estimated for each patient using exit survey data on household assets in combination with asset ownership data from the 2008 Nigeria Demographic and Health Survey.

**Results:**

Among the poorest SES group, 29% sought treatment at public health centres, 13% at pharmacies and 58% at patent medicine dealers (p < 0.01). Very few of those in the richest SES group used public health centres (4%) instead choosing to go to pharmacies (44%) and patent medicine dealers (52%, p < 0.001). During consultations with a healthcare provider, the poorest compared to the richest were significantly more likely to discuss symptoms with the provider, be physically examined and rely on providers for diagnosis and treatment rather than request a specific medicine. Those from the poorest SES group were however, least likely to request or to receive an antimalarial (p < 0.001). The use of artemisinin combination therapy (ACT), the recommended treatment for uncomplicated malaria, was low across all SES groups.

**Conclusions:**

The quality of malaria treatment is sub-optimal for all febrile patients. Having greater interaction with the provider also did not translate to better quality care for the poor. The poor face a number of significant barriers to accessing quality treatment especially in relation to treatment seeking behaviour and type of treatment received. Strategies to address these inequities are fundamental to achieving universal coverage of effective malaria treatment and ensuring that the most vulnerable people are not left behind.

## Background

Artemisinin Combination Therapy (ACT) has been the first line recommended medicine for uncomplicated malaria in Nigeria since 2005 and government treatment guidelines recommend that all febrile patients should be presumptively treated with this drug when malaria testing is not available [1]. The Nigerian government has also implemented initiatives designed to improve coverage, availability and uptake of effective malaria treatment; these initiatives include home management of malaria through training of community health workers, and supplying ACT without charge to pregnant women and children under-five attending primary health facilities [2].

Differential treatment seeking for malaria in South-East Nigeria has been reported through a number of household surveys [3-6]. These studies have found that those from poorer households attend low level and informal providers, or may not seek treatment at all [4,7,8]. These low level providers have often been associated with substandard practices including poor counselling, incorrect dosing, misdiagnosis and the use of less effective drugs [9,10]. While these studies offer an overall description of treatment seeking for febrile illness, little detail is known about the reasons for choice of treatment providers and the quality of care obtained for uncomplicated malaria across socioeconomic status groups. Given the present efforts by government and non-governmental agencies to subsidise ACT in both public and private provider outlets [11], it is important to understand the extent to which individuals of different socioeconomic status (SES) receive these antimalarials.

Quality of care is closely intertwined with treatment seeking behaviour and perceptions of quality can influence patterns of treatment seeking [12]. Quality of care as a concept has been defined in different dimensions and its elements described along the structure-process-outcome continuum [13,14]. While structural measures relate to those of the physical environment, process focuses on what happens in the interaction between the patient and the provider and outcomes typically include the consequences of care [14]. Each of these dimensions is known to be important in the context of malaria [15-17] but few have explored this in relation to SES [18]. The process of care, in particular the patient-provider interaction, is the least well understood in the context of febrile illness and merits more enquiries due to its potential influence on the outcomes of care [19].

Enhanced patient-provider interaction is characterised by extended dialogue which enables patients to disclose vital information about their health problems and providers to make more accurate diagnoses [19,20]. It provides the enabling context within which the information about the treatment regimen is communicated, which in turn can foster adherence to regimen and ultimately lead to better care outcomes [20,21]. These outcomes could be reflected in the type of treatment received, reduction of patient’s symptoms and recovery [21]. Studies show that poor patient provider interaction often arises from the existence of differential educational, socio-economic and cultural backgrounds as well as language barriers and environmental factors [22]. Individual and health system related constraints including lack of communication skills on the part of either the provider or the patient [23], high workload, and staff shortages, play prominent roles in provider-patient interaction [24]. Understanding the patient-provider interaction and the outcomes of the interaction can provide useful insight into the aspects of care process that influence delivery of quality malaria treatment to patients of different socioeconomic status.

This paper focuses on the patient-provider interaction and the outcomes of these interactions across SES groups. Data collected from patients or their caregivers as they exit public health centres, pharmacies and patent medicine dealers (PMDs) is used to describe inequities in treatment seeking, including the reasons for choice of treatment providers, detail of the interactions that took place between patient and provider and type of treatment received. The findings from this study provide insight into the type of interventions needed for achieving improvement in quality and coverage of effective malaria treatment.

## Methods

### Study setting

Formative research was undertaken in Enugu state, south-east Nigeria, to inform the selection and design of interventions to improve diagnosis and treatment of malaria among febrile patients attending public and private sector providers. This study has been described in detail elsewhere [25]. In summary, the study was undertaken in Udi, a rural local government area (LGA) and urban areas of Enugu (comprising Enugu East, Enugu South and Enugu North LGAs) between July and December 2009. Malaria is endemic in both sites and occurs throughout the year, but peaks around September to October during the rainy season. The study sites are similar in terms of language and culture. The study was undertaken at primary health centres (PHC) (which comprises of public health centres, health posts and dispensaries), pharmacies and patent medicine dealers (PMDs) also known as private medicine retailers. PHC facilities are usually staffed by community health officers, community health extension workers and less often nurses and midwives, and at these facilities treatment of uncomplicated malaria with an ACT is free of charge for pregnant women and children. Licensed pharmacists manage pharmacy shops where they dispense and sell pharmaceutical products as well as provide advice to patients [26]. PMDs are licensed to sell over the counter drugs and are a major source of malaria treatment [27]. There are no legal requirements about the education or training of PMDs, and it is accepted that they have a commercial role. At pharmacies and PMDs, patients pay the full cost of drugs. ACT was introduced to the study site in 2005 and over three-quarters (80%) of facilities were reported to have ACT in stock at the time of this study [25].

### Study design

A multi-stage cluster survey was conducted in 16 (8 communities in each site) randomly selected communities, stratified by type of facility. All PHCs in the communities were included due to their small number while pharmacies and PMDs were randomly selected with probability proportionate to size assuming that a total of 80 facilities could be visited. Respondents were febrile patients of all ages or their caregivers visiting the selected facilities during the period of the survey (July to December, 2009). Written consent from patients and caregiver was sought before determining their eligibility to participate in the survey. An individual was considered eligible if s/he reported seeking treatment for a fever or if s/he had received an ACT, were older than 6 months, and not pregnant. Individuals who were exiting a facility were assessed in turn until the patient quota of 20 patients per PHC and 14 per pharmacy and PMD was reached. These sample sizes per facility were determined for the primary study outcome, proportion of febrile patients receiving the recommended treatment for malaria [25].

### Data collection

Data were collected from eligible respondents as they exited the facility if they had given consent. Treatment may be sought for oneself, a child or another person who is not present (the latter applies only at pharmacies and PMDs). Information was obtained on general demographic details, previous treatment seeking for the illness episode, reasons for the choice of provider, and nature of interaction with the provider, including whether the provider was told about the patients’ symptoms, whether the patient was physically examined, was tested for malaria, as well as the treatment received. Respondents were also asked about asset ownership in their household (including source of drinking water, type of cooking fuel, toilet facilities and building materials). These questions on asset ownership were identical to those in the National Demographic and Health Survey conducted in Nigeria in 2008 [28].

### Statistical analysis

There were two parts to the statistical analysis. The first part of the analysis focused on the measurement of socioeconomic status. This involved comparing asset ownership of patients to the asset ownership of individuals living in comparable areas in South-East Nigeria (from the Demographic and Health Survey, DHS) [28]. This was possible since the questions on asset ownership were the same as those that the DHS asked of survey respondents. This approach was adopted in order to describe the SES of patients with reference to the local population (rather than relative to the sub-group that sought treatment).The approach used principal components analysis to compute weights for the ownership of a defined list of assets using DHS data collected in urban areas of South-East Nigeria in 2008 [29,30]. A wealth score was generated for individuals in the DHS data set, where the wealth score is the sum of the weights for those assets that an individual owns. Individuals in the DHS population were then ranked by their wealth score and divided into five groups, and this determined the cut-off values of the wealth score for each quintile. The next step was to estimate the wealth scores of each patient in the exit survey, using the weights derived from the DHS population. Patients were then assigned to one of the socioeconomic quintiles based on their wealth score and the previously determined cut-off values [29]. DHS data was selected to be representative of the local population, though we acknowledge differences in the geographic areas and timing of data collection. As there were relatively few exit survey respondents from the poorest 40% of the population the poorest two quintiles were combined into one SES group. The SES groups were then poorest quintile (Q1 and Q2), 3rd quintile (Q3), 4th quintile (Q4) and richest quintile (Q5).

The second part involved describing patient characteristics and geographic distribution as well as treatment seeking behaviour, health care interaction and treatment received. Specific variables used to describe treatment seeking behaviour included the timing of treatment seeking, type of provider visited and reasons for the choice of provider. For describing the patient-provider interaction, variables included whether the patient told the health worker about symptoms, whether health worker asked follow up questions about patients illness, physically examined patient, took patients’ temperature, tested patient for malaria and whether patient requested a specific medicine. Variables to describe outcome were the type of treatment received and whether patients received an ACT in the correct dose and knew the regimen [25].

Relative to the local population, the patient exit data were analysed by SES group to describe the study population, treatment seeking behaviour, and health care interaction including treatment received. The percentages reported are population-average estimates, which have been adjusted for the study design by identifying different probabilities of selection, clustering and stratification [30]. Outcomes by SES group were compared using the Rao and Scott chi-squared correction [30].

### Ethical approval

Ethical approval for this study was obtained from the University of Nigeria ethics review board (UNTH/CSA.329) and London School of Hygiene and Tropical Medicine, UK (approval 5429).

## Results

Data were collected from 100 health facilities using a pre-tested interviewer-administered questionnaire and the analysis is based on exit data collected from 1642 patients, having excluded 33 pregnant women and 28 children under six months that did not meet the eligibility criteria. Characteristics of patients included in the study are presented in Table 1. Significant variations in all characteristics (except gender) were observed between the SES groups. Those patients from the poorest quintile were more likely to reside in a rural location (p < 0.001), be less than 5 years (p = 0.005), and have a lower level of education (or with a caregiver with a lower level of education if the patient was a child) (p < 0.001).

**Table 1:** Characteristics of patients for whom treatment was sought*

	**Poorest 40%**	**Third 20%**	**Fourth 20%**	**Richest 20%**	**All**	**P-value**
	n (%) [95% CI]*	n (%) [95% CI]*	n (%) [95% CI]*	n (%) [95% CI]*	n (%) [95% CI]*	
	**N = 362**	**N = 292**	**N = 423**	**N = 565**	**N = 1642**	
**Area of residence**						
Enugu (urban)	54 (30.9) [19.2-45.8]	150 (79.4) [70.3-86.3]	346 (93.1) [88.85.8]	512 (96.6) [94.0-98.1]	1062 (87.9) [85.2-90.2]	<0.001
Udi (rural)	308 (69.1) [54.2-80.8]	142 (20.6) [13.8-29.7]	77 (7.0) [4.3-11.2]	53 (3.4) [1.9-6.0]	580 (12.1) [9.8-14.8]	
**Gender** ^**a**^						
Male	161 (45.2) [36.3-54.5]	140 (53.4) [40.0-66.5]	228 (55.5) [46.0-64.5]	313 (57.9) [51.1-64.4]	842 (55.5) [51.7-59.3]	0.482
Female	195 (54.8) [45.5-63.7]	147 (46.6) [33.5-60.2]	191 (44.5) [35.5-48.9]	245 (42.1) [35.6-48.9]	778 (44.5) [40.8-48.3]	
**Age group**						
>15 yrs	196 (64.0) [54.5-72.5]	176 (71.4) [63.2-78.4]	319 (83.8) [76.7-89.0]	407 (75.8) [69.6-81.1]	1098 (76.7) [72.44-80.5]	0.005
5 to 15 yrs	69 (17.2) [12.4-23.3]	50 (13.8) [8.7-21.1]	46 (8.0) [5.1-12.3]	79 (14.4) [10.6-19.2]	244 (12.5) [10.1-15.5]	
<5 yrs	97 (18.8) [12.5-27.4]	66 (14.8) [8.9-23.6]	58 (8.2) [4.7-14.0]	79 (9.8) [6.7-14.0]	300 (10.7) [8.1-14.0]	
**Education level of patient (or caregiver)** ^**b**^						
Primary	195 (52.3) [43.4-61.1]	86 (29.4) [20-8-39.9]	54 (12.1) [8.0-17.8]	45 (7.3) [4.5-11.5]	380 (15.4) [12.4-18.9]	<0.001
Secondary	132 (41.5) [32.8-50.9]	140 (41.8) [31.7-52.6]	192 (43.8) [35.0-53.0]	218 (37.2) [29.9-45.1]	682 (40.2) [34.9-45.7]	
Tertiary	18 (6.2) [2.4-15.2]	57 (28.8) [21.4-37.5]	171 (44.1) [35.3-53.4]	292 (55.6) [47.2-63.8]	538 (44.5) [38.9-50.1]	

*Population averaged percentages which have been adjusted for the survey design.

Notes: (^a^)missing 22 responses; (^b^)missing 42 responses.

### Inequities in treatment seeking behaviour

Around half (47%) of those patients seeking treatment were from the richest 20% of the population, while about one in ten patients were from the poorest 40% of the population. There was evidence that a significantly longer duration had elapsed between onset of symptoms and presentation at health facilities for treatment among those in the poorest SES groups, compared to richer SES groups (p = 0.044). Those in the poorer SES groups were however more likely to have previously sought treatment for their current illness prior to seeking treatment at the study facilities (p = 0.002). Of the 469 patients who had previously sought treatment, most had done so at a PMD across all SES groups though the richer SES groups also sought treatment at pharmacies (p = 0.006). Only 6% received an ACT in their previous treatment while 39% received an antimalarial (no significant difference across SES group) (Table 2).

**Table 2:** **Inequalities in treatment seeking behaviour**

	**Poorest 40%**	**Third 20%**	**Fourth 20%**	**Richest 20%**	**All**	**P-value**
	n (%) [95% CI]*	n (%) [95% CI]*	n (%) [95% CI]*	n (%) [95% CI]*	n (%) [95% CI]*	
	**N = 362**	**N = 292**	**N = 423**	**N = 565**	**N = 1642**	
**Number of days since start of symptoms** ^**c**^						
Same day	31 (11.4) [5.6-22.1]	51 (27.2) [16.6-41.3]	77 (24.2) [17.4-32.8]	89 (17.8) [13.5-22.9]	248 (20.6) [16.1-25.9]	
1 day	61 (16.3) [(9.8-25.8]	64 (22.4) [15.6-31.0]	82 (17.2) [10.9-26.0]	123 (22.9) [16.6-30.6]	330 (20.5) [16.0-25.9]	0.044
2 days	61 (15.0) [9.5-22.8]	54 (19.3) [11.5-30.7]	80 (21.6) [16.1-28.4]	126 (21.1) [17.9-27.2]	321 (21.1) [18.1-24.4]	
3-5 days	121 (33.4) [24.7-43.5]	93 (24.8) [17.2-34.2]	118 (23.6) [16.8-32.0]	154 (25.3) [19.6-31.6]	486 (25.3) [21.2-29.7]	
6+ days	88 (23.9) [16.2-33.8]	29 (6.4) [3.1-12.6]	64 (13.4) [8.4-20.7]	73 (12.6) [8.6-16.6]	254 (12.6) [9.3-16.9]	
**First time treatment was sought for illness episode** ^**d**^						
Yes	202 (60.7) [51.3-69.4]	192 (80.9) [69.8-88.6]	327 (82.9) [74.9-88.7]	444 (81.2) [75.8-85.7]	1165 (80.1) [75.2-84.2]	0.002
No	160 (39.3) [30.7-48.7]	98 (19.1) [11.4-30.2]	93 (17.1) [11.3-25.1]	118 (18.8) [14.3-24.2]	469 (19.9) [15.8-24.9]	
**Of those that previously sought treatment for this illness episode what type of provider was visited?**						
Public hospital	13 (9.0 [2.7-26.4]	9 (13) [3.2-40.2]	4 (1.0) [0.2-5.2]	10 (9.4) [3.4-23.3]	36 (7.6) [3.5-15.5]	0.006
Primary health facility	15 (5.6) [3.0-10.2]	5 (3.5) [1.5-8.0]	11 (7.5) [2.7-18.8]	7 (1.2) [0.4-3.5]	38 (4.0) [2.2-7.2]	
Pharmacy	3 (6.0) [0.8-33.5]	3 (3.0) [0.6-14.4]	14 (19.7) [7.0-44.3]	31 (37.0) [25.2-50.6]	51 (22.5) [14.4-33.4]	
Patent medicine dealer	94 (62.7) [45.7-77.0]	53 (57.0) [39.9-72.5]	49 (57.3) [36.9-75.5]	38 (36.3) [25.2-50.6]	234 (49.1) [38.8-59.5]	
Other	32 (16.7) [8.5-30.0]	25 (23.5) 10.0-45.7]	12 (14.6) [6.9-28.2]	22 (16.1) [9.2-26.8]	91 (16.8) [11.8-23.4]	
**Of those that previously sought treatment, what treatment was obtained? (n = 469)**						
Any AM	43 (27.4) [16.5-42.0]	30 (29.3) [17.1-45.6]	39 (43.9) [28.7-60.3]	48 (43.0) [27.9-59.6]	160 (38.9) [29.7-49.1]	0.265
ACT	6 (2.7) [1.1-6.6]	1 (1.0) [0.3-3.1]	9 (9.8) [3.2-27.0]	6 (5.8) [1.8-17.1]	22 (5.8) [2.6-12.4]	0.160
Antipyretic	78 (39.4) [27.4-52.8]	51 (43.4) [29.8-58.4)	46 (42.8) [30.3-56.2]	59 (49.7) [35.9-63.4]	234 (45.4) [37.2-53.9]	0.581
Antibiotic	14 (7.0) [3.7-12.8]	10 (10.6) [2.8-32.9]	5 (4.5) [1.1-16.9]	9 (7.0) [2.4-18.8]	38 (6.8) [3.5-12.6]	0.715
**Type of provider for current treatment**						
Public facility	144 (28.8) [19.6-40.2]	116 (13.5) [9.2-19.5]	103 (5.5) [4.4-7.2]	103 (4.3) [2.3-8.0]	466 (7.9) [6.5-9.5]	<0.001
Pharmacy	14 (13.0) [4.8-30.5]	38 (23.8) [10.8-44.5]	111 (34.1) [17.5-55.7]	199 (44.0) [27.4-62.1]	362 (35.6) (21.9-52.2]	
Patent Medicine Dealer	204 (58.3) [43.4-71.7]	138 (62.7) [42.4-79.3]	209 (60.4) [39.5-78.1]	263 (51.8) [34.2-68.9]	814 (56.5) [40.6-71.1]	
**Reasons given for the choice of provider** ^**e**^						
Have used before	263 (67.0) [55.4-76.9]	178 (52.7) [35.4-69.3]	218 (47.3) [37.2-57.5]	316 (57.1) [48.7-65.1]	975 (54.2) [46.7-61.6]	0.103
Convenient location	155 (43.6) [32.8-55.1]	154 (59.3) [45.4-71.8]	238 (61.1) [52.1-69.3]	299 (52.5) [43.9-61.0]	846 (55.4) [48.9-61.8]	0.1041
Good reputation	100 (26.0) [18.6-35.1]	110 (43.6) [30.3-57.9]	187 (46.4) [38.4-54.7]	277 (54.2) [45.1-63.0]	674 (48.1) [40.5-55.7]	0.002
Availability of drugs	126 (33.5) [23.2-45.8]	116 (41.8) [30.4-54.1]	202 (48.9) [34.5-63.5]	231 (48.8) [37.2-60.6]	675 (46.7) [37.4-56.6]	0.280
Inexpensive	111 (27.5) [19.3-37.5]	86 (21.5) [14.8-30.2]	94 (15.1) (9.2-24.0)	93 (12.8) [8.8-18.3]	384 (15.9) [12.1-20.7]	0.021
Qualification of staff	65 (13.9) [7.5-24.3]	79 (17.6) [9.4-30.6]	97 (19.3 (11.6-30.3)	65. (9.2) [5.4-15.3]	306 (13.9) [9.4-20.1]	0.022
**Travel time (minutes)** ^**f**^						
0-15	149 (46.3) [35.6-57.4]	169 (74.2) [63.4-82.6]	290 (82.2) [73.7-88.4]	395 (76.1) [68.6-82.2]	1003 (75.3) [69.7-80.1]	<0.001
16-30	107 (27.6) [20.3-36.4]	64 (16.4) [10.6-24.5]	67 (12.6) [7.6-20.3]	110 (17.1) [11.9-24.0]	348 (16.5) [12.7-21.1]	
31-45	39 (9.3) [5.1-16.3]	24 (5.2) [1.8-14.0]	23 (3.5) [1.7-7.1]	23 (4.5) [2.2-8.9]	109 (4.6) (3.1-7.0]	
46-60	22 (6.2) [3.6-10.4]	13 (2.8) [1.0-7.0]	6 (0.4) [0.2-1.0]	12 (1.5) [0.6-3.9]	53 (1.7) [1.1-2.8]	
>60	32 (10.7) [4.2-24.5]	6 (1.5) [0.3-8.0]	10 (1.3) [0.4-4.2]	5 (0.8) [0.2-3.6]	53 (1.9) [0.9-3.7]	

*Population averaged percentages and corresponding 95% confidence intervals, which have been adjusted for the survey design.

Notes: (^c^)missing 3 responses; (^d^)missing 8 responses (^e^)unprompted and multiple responses were possible; (^f^)missing 15 responses.

For current treatment, PMDs were again widely used by all SES groups, though PHC facilities were also frequently used by those of lower SES while pharmacies mostly served those from richer SES (p < 0.001). Those in the poorest SES groups were more likely to travel further to seek treatment (p < 0.001) (Table 2).

With respect to reasons for the choice of provider, providers’ reputation, cost and staff qualifications/experience were important determinants that differed significantly between the SES groups. Provider reputation was more likely to be a consideration for those in richer SES groups (p = 0.002) while qualification of staff (p = 0.022) and cost of treatment were more often noted by those from the poorer SES groups (p = 0.021).

### Inequities in the patient-provider interaction and treatment received

Significant differences were revealed in the nature of patient-provider interaction, with those of lower SES more reliant on the provider to diagnose their illness. For example, the poorest were more likely to have told the provider about their symptoms (p < 0.001), be asked follow up questions about their illness (p = 0.011), undergo a physical examination (p = 0.031), and have their temperature taken (p = 0.007). Across all SES groups less than 1% of patients were tested for malaria using either microscopy or RDT.

About 79% of patients seeking treatment for a fever received an antimalarial and the proportion differed significantly by SES (p < 0.001). About 22% received an ACT and 40% received SP, a smaller number, 13% received monotherapy and about 50% received an antipyretic no significant differences were observed in these results across SES.

Those in the richer SES groups were more likely to receive an antimalarial (p < 0.001) and to have requested a specific medicine from the provider (p < 0.001) with those from the lower SES groups more dependent on the provider to recommend medicines. About 61% of all patients reported asking for a specific medicine during their interaction with a provider, with 89% requesting an antimalarial and 25% requesting an ACT. There was no significant difference across SES groups in the type of medicine requested or the type of treatment received. More than half of those receiving an ACT across all SES, did so in the correct dose (p = 0.716) and knew the treatment regimen (p = 0.655), but there were no significant variations by SES (Table 3).

**Table 3:** **Inequalities in patient-provider interaction and treatment received**

	**Poorest 40%**	**Third 20%**	**Fourth 20%**	**Richest 20%**	**All**	**P-value**
	n (%) [95% CI]*	n (%) [95% CI]*	n (%) [95% CI]*	n (%) [95% CI]*	n (%) [95% CI]*	
	**N = 362**	**N = 292**	**N = 423**	**N = 565**	**N = 1642**	
**Nature of interaction**						
Told HW about fever^(a)^	312 (75.2) [61.0-85.5]	220 (54.7) [42.1-66.6]	248 (44.1) [36.3-52.2]	255 (36.1) [25.9-47.7]	1035 (44.3) [37.3-51.4]	<0.001
HW asked questions about illness^(b)^	252 (59.9) [47.8-70.9]	184 (41.7) [27.9-56.9]	211 (36.2) [28.6-44.6]	228 (29.7) [20.2-41.3]	875 (35.7) [29.1-43.1]	0.011
Patient was physically examined	116 (21.9) [13.6-33.2]	101 (14.8) [9.2-22.9]	85 (7.6) [4.4-13.1]	101 (9.8) [5.7-16.2]	403 (10.8) [7.9-14.5]	0.031
Patient had temperature taken	98 (16.8) [9.7-27.5]	69 (7.6) [4.6-12.2]	49 (3.4) [2.0-5.7]	54 (4.4) [1.9-9.8]	270 (5.5) [3.9-7.8]	0.007
Patient was tested for malaria	7 (1.0) [0.6-1.7]	6 (1.0) [0.7-1.4]	3 (0.2) [0.1-0.6]	11 (0.8) [0.3-2.3]	27 (0.7) [0.3-1.3]	0.076
Requested a specific medicine	54 (23.3) [13.4-37.4]	85 (49.0) [36.8-61.4]	215 (63.6) [54.7-71.7]	328 (68.1) [57.2-77.4]	682 (60.5) [53.7-66.9]	<0.001
**For those that requested specific medicine, type of treatment requested N = 676**						
Antimalarial^(c)^	27 (69.5) [40.2-88.5)	69 (81.3) [56.6-93.5)	195 (93.7) [86.4-97.2]	295 (89.1) [82.1-93.5)	586 (89.1) [83.1-93.1]	0.057
ACT^**(c)**^	6 (26.2) [5.9-66.6]	12 [13.8) [5.6-30.1]	46 (24) [14.7-36.7)	85 (27.0) [18.6-37.5]	149 (24.5) [17.7-32.9]	0.458
Artesunate monotherapy^(c)^	2 (3.9) [0.5-23.9]	11 (13.8) [5.6-30.4)	39 (18.7) [12.3-27.3]	61 (18.5) [13.4-25.1]	113 (17.6) 13.6-22.4)	0.452
Sulphadoxine Pyrimethamine^(c)^	13 (32.1) [11.7-62.8]	35 (41.8) [25.0-60.7]	89 (43.4) [33.9-53.4]	126 (37.9) [28.3-48.5]	263 (39.9) [33.0-47.3]	0.741
Other type of antimalarial^(c)^	6 (7.3) [2.1-22.1]	11 (11.9) [4.8-26.6]	21 (7.7) [3.8-15.0]	23 (5.7) [3.3-9.5]	61 (7.1) [4.9-10.2]	0.395
Antibiotic^(c)^	7 (7.8) [2.7-20.6]	5 (8.7) [2.0-30.9]	8 (5.0) [1.9-12.1]	21 (6.1) [3.0-12.0]	41 (6.1) [3.5-10.4]	0.775
Antipyretic^(c)^	19 (31.7) [16.1-52.8]	38 (46.3) [30.4-62.9]	101 (45.9) [35.8-56.4]	162 (50.0) [40.8-59.2]	320 (47.7) [41.1-54.5]	0.563
**For those that requested specific medicine, treatment received N = 586**						
Antimalarial	22 (93.8) [80.8-98.2]	64 (96.5) [86.1-99.2]	190 (98.0) [92.5-99.5]	274 (93.4) [82.0-97.8]	550 (95.3) [89.8-97.9]	0.265
ACT	5 (96.7) [74.5-99.7]	9 (74.4) [22.5-96.7]	43 (95.7) [79.0-99.2]	68 (83.7) [54.3-95.7]	125 (87.3) [67.6-95.8]	0.235
Artesunate monotherapy	1(77.9) [12.6-98.9]	10 (87.8) [27.4-99.3]	37 (94.2) [67.4-99.2]	49 (85.0) [57.2-91.3]	97 (85.0) [68.8-93.6]	0.316
Sulphadoxine Pyrimethamine	11 (94.4) [71.9-99.1]	32 (97.2) [86.3-99.5]	86 (96.3) [83.0-99.3]	120 (96.2) [86.8-99.0]	249 (96.3) [91.4-98.5]	0.948
Other type of antimalarial^(c)^	3 (66.1 [23.5-92.5]	9 (81.2) [29.4-97.8)	15 (64.7) [30.8-88.3]	17 (75.6) [54.9-85.2]	44 (72.5) [54.9-85.2]	0.789
Antibiotic^(c)^	5 (70.7) [30.7-92.9]	5 (100)	7 (90.3) [41.5-99.2]	19 (89.2) [42.5-98.9]	36 (90.6) [64.7-98.1]	0.704
Antipyretic^(c)^	17 (93.2) [70.3-98.8]	36 (89.9) [65.9-97.6]	97 (94.4) [81.1-98.5]	154 (96.2) [90.6-98.5]	304 (94.9) [88.6-97.8]	0.444
**Treatment received for all febrile patients N = 1642**						
Antimalarial	179 (58.1) [46.01-69.3]	184 (71.9) [60.5-81.1]	339 (87.0) [80.7-91.5]	424 (80.1) [72.1-86.2]	1126 (79.3) [74.5-83.4]	<0.001
ACT	38 (11.3) [4.6-24.9]	58 (18.1) [10.3-29.8]	91 (22.0) [15.1-30.8]	128 (25.7) [19.3-33.4]	315 (22.4) [17.0-28.8]	0.100
Artesunate monotherapy	10 (3.5) [1.3-9.2]	22 (9.9) [4.8-19.1]	44 (13.2) [8.8-19.3]	82 (16.2) [12.1-21.3]	158 (13.4) [10.6-16.7]	0.053
Sulphadoxine Pyrimethamine	84 (30.7) [20.3-43.6]	82 (35.6) [24.8-48.2]	178 (45.8) [36.4-55.5]	192 (34.6) [26.4-43.9]	536 (37.9) [31.9-44.3]	0.117
Other type of antimalarial	47 (12.6) [7.3-20.9]	27 (10.9) [6.1-18.7]	29 (6.8) [3.6-12.4]	42 (7.4) [4.7-11.5]	145 (8.2) [6.1-10.7]	0.327
Antibiotic	76 (16.0) [10.7-23.3]	39 (10.8) [5.6-19.8]	27 (6.8) [3.8-11.7]	51 (8.4) [5.6-12.5]	193 (8.8) [6.8-11.3)	0.138
Antipyretic	226 (59.4) [49.3-68.7]	167 (56.7) [44.1-68.4]	211 (52.3) [42.6-61.8]	270 (50.8) [43.3-58.3]	875 (52.8) [47.7-57.8]	0.584
**For those that received ACT**						
Received ACT in correct dose	25 (56.5) [17.4-88.9]	47 (77.0) [53.2-90.8]	48 (62.4) [42.6-78.7]	81 (67.7) [45.3-84.2]	201 (67.0) [53.3-78.2]	0.716
Patient knows regimen	20 (48.1) [15.2-82.7]	41 (72.4) [47.8-88.2]	41 (54.3) [31.3-75.6]	67 (59.8) [36.4-79.5]	169 (59.5) [43.7-73.5]	0.655

*Population averaged percentages and corresponding 95% confidence intervals, which have been adjusted for the survey design. (^a^)Missing one response (^b^)Missing two responses (^c^)Missing 11 responses.

## Discussion

This paper has presented new information about inequities in the nature of health care interaction and quality of treatment received for febrile illness in South-East Nigeria. Several inequities in treatment seeking for febrile illness were identified in this study. The interval between the onset of symptoms and treatment seeking was significantly greater in the poorest SES group compared to the richest; the poor were also less likely to be seeking treatment for the first time which suggests that previous treatment may have been ineffective. The finding that the poor were more likely than the rich to attend public facilities rather than pharmacies was consistent with reported reasons for choice of provider. For the poorest, the choice of provider was heavily influenced by cost. Public facilities offer malaria treatment at no cost for pregnant women and children while private facilities charge higher prices compared to public facilities [31]. These patterns of treatment seeking are contrary to what has been reported in earlier studies in South-East Nigeria where the poor were found to use lower level providers (traditional healers, PMDs) while the rich relied more heavily on public facilities [7]. Though this difference may be partly explained by the age profile of patients since the proportion of children attending public facilities was significantly higher among the poorest SES group [25].

There were also important differences in the nature of the patient-provider interaction across socioeconomic groups. Differential health care interactions have been reported elsewhere suggesting a tendency for providers to have better interactions with those of higher SES [32] due to the presumption that the poor are less well educated and thus less able to understand the information given by the provider. In contrast, we found that those in the lower SES groups were more likely to discuss symptoms with providers, be examined and rely on the provider to recommend treatment. However, this is likely to be influenced by the fact that it was more common for the lower SES groups to seek treatment at public facilities. In other words, some of these differences may reflect the type of provider at which treatment was sought [25]. The nature of interaction does not, however, explain the treatment received given that overall uptake of ACT was low from all providers.

It was also surprising to find that only 11% of febrile patients in the poorest SES received an ACT as recommended, when 29% of respondents from this SES group attended a public facility and depended on the provider to recommend treatment. On this point, it is important to note that very few tests (less than 1% of patients) were carried out, and the malaria treatment guidelines advise presumptive treatment of malaria in the absence of a malaria test [1]. It is, therefore, unclear why so few patients, especially those attending public facilities received an ACT. Other studies have highlighted factors that may influence providers’ decisions to give (or not) an ACT including fear of stock outs due to inconsistent supply of ACTs [33] and patients preferences [25].

Although it was found that those of the richest SES group were more likely to request an antimalarial, only 27% requested an ACT, and far more requested Sulphadoxine Pyrimethamine (SP), which is no longer recommended for treating malaria due to extensive resistance [1]. Similar problems have previously been highlighted, especially at pharmacies and PMDs where patients commonly request specific medicines that are most often not the recommended ones [25,34]. These suggest there are also widespread demand side problems with the uptake of ACT which could be due to the low awareness that ACT is the recommended treatment and the comparative cost of ACT [35]. The cost of ACTs averages at $3.6, about three times the cost of SP thus a 2 to 3 days income will be needed to treat a malaria case with an ACT in Nigeria where over 50% live below $2 per day [36].

Overall our findings highlight socioeconomic inequities in timing of treatment seeking for febrile illness and significant problems with the uptake of ACT. Though there are no significant differences in the uptake of ACTs, it cannot be conclusively stated that there is equity in the use of ACTs given the low uptake by all SES groups but especially the poorest. Another study found equity in the use of ACTs among respondents following a free distribution exercise in south-east Nigeria though the two studies employed different methodologies [37]. Our findings also suggest that improving the process of care may not lead to better quality care, highlighting the need for further exploration of factors that constitute barriers to uptake of quality malaria treatment. These findings suggest minimal progress towards achieving timely and equitable coverage of effective malaria treatment in South-East Nigeria. These results are in line with other studies from Sub -Saharan Africa which show better-off individuals are significantly more likely to obtain antimalarials, and in particular to obtain effective antimalarials [38,39]. The findings from this study complement existing literature by providing new insights into provider-patient interactions, including which treatments were requested and received by different SES groups. These findings are important for a number of reasons. First, there is limited evidence on the process of care, including the specific interactions that take place between febrile patients and malaria treatment providers and how they influence outcomes of care for febrile illness, and a better understanding of the care process is important for overall quality improvements in malaria treatment. Second, in the current context of ACT subsidies and targeting for the poor, a body of evidence on the extent and potential barriers of uptake by different socioeconomic groups is necessary to throw light on progress towards effective coverage of recommended antimalarials and where more effort is required. Lastly, by using the socioeconomic quintiles that apply to the general population, the findings of this study can inform interventions to improve malaria treatment in South-East Nigeria.

One limitation of the study is that the technique used to determine the SES of the patients attending facilities is based on an assumption that the household data used to generate the factor weights is representative of the local population in the study sites at the time of the exit survey. The choice of reference group may affect the precision of the factor weights, and therefore the cut-off values between different SES categories. However, given the substantial difference by SES group, choice of reference group is unlikely to affect the overall trends reported. A second limitation, already noted, concerns the fact that the respondents seeking treatment tended to be from higher socio-economic groups with individuals from the poorest and second poorest quintiles under-represented in the survey relative to their population share, which restricts generalisation, in addition, we did not explore the extent to which patient’s choice of antimalarials is a function of their income, this may have helped to explain the low uptake of ACTs.

In conclusion, the quality of malaria treatment is sub-optimal for all febrile patients, but worse for the poorest socioeconomic groups. The fact that the poorest SES group were more likely to seek treatment at PHC facilities and had greater interaction with the provider did not translate to better quality care. Our findings highlight the need for strategies that will improve patient’s demand for the recommended treatment and encourage provider adherence to treatment using ACT. These strategies are fundamental to achieving universal health coverage and ensuring that the most vulnerable people are not left behind.
